# Detailed Comparison of Westergren ESR and Automated 18°C ESR Using the YHLO VISION ESR Analyzer: A Comprehensive Assessment in an Indian Cohort

**DOI:** 10.7759/cureus.102548

**Published:** 2026-01-29

**Authors:** Kartavya K Verma, Girish S Kshatriya, Himanshu Upadhyay

**Affiliations:** 1 Pathology, Shri Rawatpura Sarkar Institute of Medical Sciences and Research, Raipur, IND; 2 Transfusion Medicine, Late Baliram Kashyap Memorial Government Medical College, Jagdalpur, IND; 3 Pathology, HLL Lifecare Limited, All India institute of Medical Sciences, Raipur, IND

**Keywords:** 18°c esr, automated esr, esr, manley method esr, westergren method

## Abstract

Background

Erythrocyte sedimentation rate (ESR) is a widely used, simple, and cost-effective test for diagnosing and monitoring conditions. Although the Westergren method is the gold standard, it has limitations like long processing times, infection risks, and a requirement for citrated blood samples. Automated ESR methods have been developed to overcome these issues.

Materials and methods

Blood samples were obtained from 4,000 patients who visited the central laboratory for ESR testing after providing informed consent. Each sample was first tested for ESR using the YHLO VISION ESR V4.0 Analyzer (Shenzhen YHLO Biotech Co., Ltd., Shenzhen, China) and subsequently analyzed with the manual Westergren method. The analysis included calculations of mean and standard deviation, Bland-Altman agreement assessment, linear regression, and Mann-Whitney U tests to evaluate the relationship between the two methods.

Result

Using data from 4,000 patient specimens, results demonstrated good agreement between methods with 94.65% of measurements within acceptable limits, a strong positive linear correlation (r = 0.9809, R² = 0.9621), and a systematic bias of 4.95 mm/h. Both methods effectively distinguished between patients with and without clinical evidence of elevated ESR (p < 0.001).

Conclusions

The 18°C measurement method provides a viable alternative with high clinical reliability and potential for cost-effectiveness.

## Introduction

The erythrocyte sedimentation rate (ESR) is a blood test used to assess various pathological conditions, including systemic inflammation [1]. The sedimentation process follows a sigmoidal pattern and includes three stages: the initial lag phase, the sedimentation or acceleration phase, and the packing or compaction phase [2]. Under low-shear flow, erythrocytes dispersed in plasma form rouleaux via interactions with inflammatory proteins such as fibrinogen and immunoglobulins and grow into aggregates over time, thereby increasing the sedimentation rate [2,3].

Since the Westergren method, the standardized international reference for ESR measurement, is a manual process that takes approximately one hour, many alternative methods and automated analyzers have been introduced to reduce testing time and enhance practicality [4]. This technique enables rapid estimation of ESR by analyzing the aggregation behavior of red blood cells in a blood sample, typically within a very short time frame. It is integrated into modern diagnostic approaches for assessing inflammation and related conditions, yielding faster results than traditional ESR measurement methods [5]. Syllectometry yields aggregation metrics derived from a syllectogram, which is a waveform of transmitted or reflected light intensity generated by the formation of red blood cell clusters [6]. However, ESR values obtained by this rapid measurement method have been reported to differ from those obtained by the Westergren method due to hematocrit [7]. The International Council for Standardization in Hematology (ICSH) advises careful consideration of method-specific variations [8]. This research aimed to compare two ESR techniques, specifically, the YHLO VISION ESR analyzer V4.0 (Shenzhen YHLO Biotech Co., Ltd., Shenzhen, China) and the traditional modified manual Westergren method, by evaluating their level of agreement and examining the correlation of the ESR values they produce.

## Materials and methods

This study was conducted on samples received at the central lab from January 2025 to July 2025 for ESR estimation. Blood samples were collected in ethylenediaminetetraacetic acid (EDTA) tubes and analyzed using the YHLO VISION ESR analyzer, which measures ESR at 18°C using the Manley method. Additionally, samples were obtained in ESR tubes for the conventional Westergren method (Desco Medical India Pvt. Ltd., Delhi, India). Concurrently with the ESR analysis, patient clinical histories of inflammatory conditions were documented, and C-reactive protein (CRP) levels were measured. Electronic medical records by two independent evaluators using a predefined checklist including presence of symptoms (fever, constitutional symptoms, arthralgias, etc.) at or near the time of ESR testing; documented clinical diagnoses related to inflammatory, infectious, or malignant conditions; physical examination findings consistent with inflammation; supporting laboratory findings (elevated CRP, positive rheumatoid factor, etc.); and imaging or tissue diagnostic confirmation when applicable. However, the factors affecting ESR are numerous, so it's not possible to eliminate all confounding factors. The data were subsequently analyzed using SPSS Statistics version 31 (IBM Corp. Released 2025. IBM SPSS Statistics for Windows, Version 31.0. Armonk, NY: IBM Corp.), and comprehensive statistical analyses were conducted.

Patient selection and inclusion criteria

Following approval from the Institutional Ethics Committee of Shri Rawatpura Sarkar Institute of Medical Sciences and Research (approval number: SRIMSR/IEC/2025/03), all patients who presented to the Shri Rawatpura Sarkar Institute of Medical Sciences and Research laboratory for a complete blood count with ESR estimation during the study period from January 2025 to July 2025 were included in this investigation.

Statistical analysis

The statistical analysis employed multiple complementary methodological approaches to comprehensively evaluate the agreement and correlation between the two measurement techniques.

Bland-Altman agreement analysis was performed as the gold standard method for assessing concordance between measurement techniques in clinical laboratory settings. The mean difference (bias) was calculated as the mean difference between ESR values obtained by the Westergren method and ESR values measured at 18°C by the automated analyzer. Limits of agreement (LoA) were established using the formula: bias ± 1.96 × SD (difference). The percentage of measurements within the LoA was calculated to assess overall agreement, and the correlation between mean values and differences was evaluated using Pearson's correlation coefficient to identify any proportional bias.

The Mann-Whitney U test was employed to evaluate whether systematic differences exist between the two measurement methods. Given the non-normal distribution of the data as determined by preliminary testing, the non-parametric Mann-Whitney U test was selected as the appropriate statistical approach. A two-sided test with significance threshold α = 0.05 was applied. The U-statistic and corresponding p-value were calculated, and the effect size was estimated using the rank-biserial correlation. A complementary Wilcoxon signed-rank test for paired comparison was also conducted to corroborate the findings.

Linear regression was used to predict the relationship between the two measurement methods. The regression model was specified as ESR₁₈°C by automated analyzer = β₀ + β₁ × ESR Westergren method. Least-squares estimation was employed to determine the regression coefficients. Goodness-of-fit was evaluated using R² and adjusted R². Ninety-five percent confidence intervals for the regression slope were established, and both root mean square error (RMSE) and residual analysis were performed to assess model precision and validity.

Subgroup analyses were conducted to assess method concordance across clinically relevant categories. Analysis by clinical condition status compared ESR values in patients with and without clinical evidence of elevated ESR. Stratification by CRP levels was performed to evaluate whether inflammatory marker intensity influenced method concordance, specifically comparing CRP >10 mg/L versus <5 mg/L. Gender-stratified analysis was also conducted by comparing ESR values between male and female participants.

Data quality and normality assessment were performed using the Shapiro-Wilk test to assess whether the data from both measurement methods followed a normal distribution. The results of normality testing informed the selection between parametric and non-parametric statistical tests for subsequent analyses.

## Results

A total of 4,000 blood samples were assessed in this investigation. The demographic distribution showed that males accounted for 2,926 participants (73.2%), whereas females accounted for 1,074 participants (26.8%).

Bland-Altman agreement analysis

**Figure 1:**
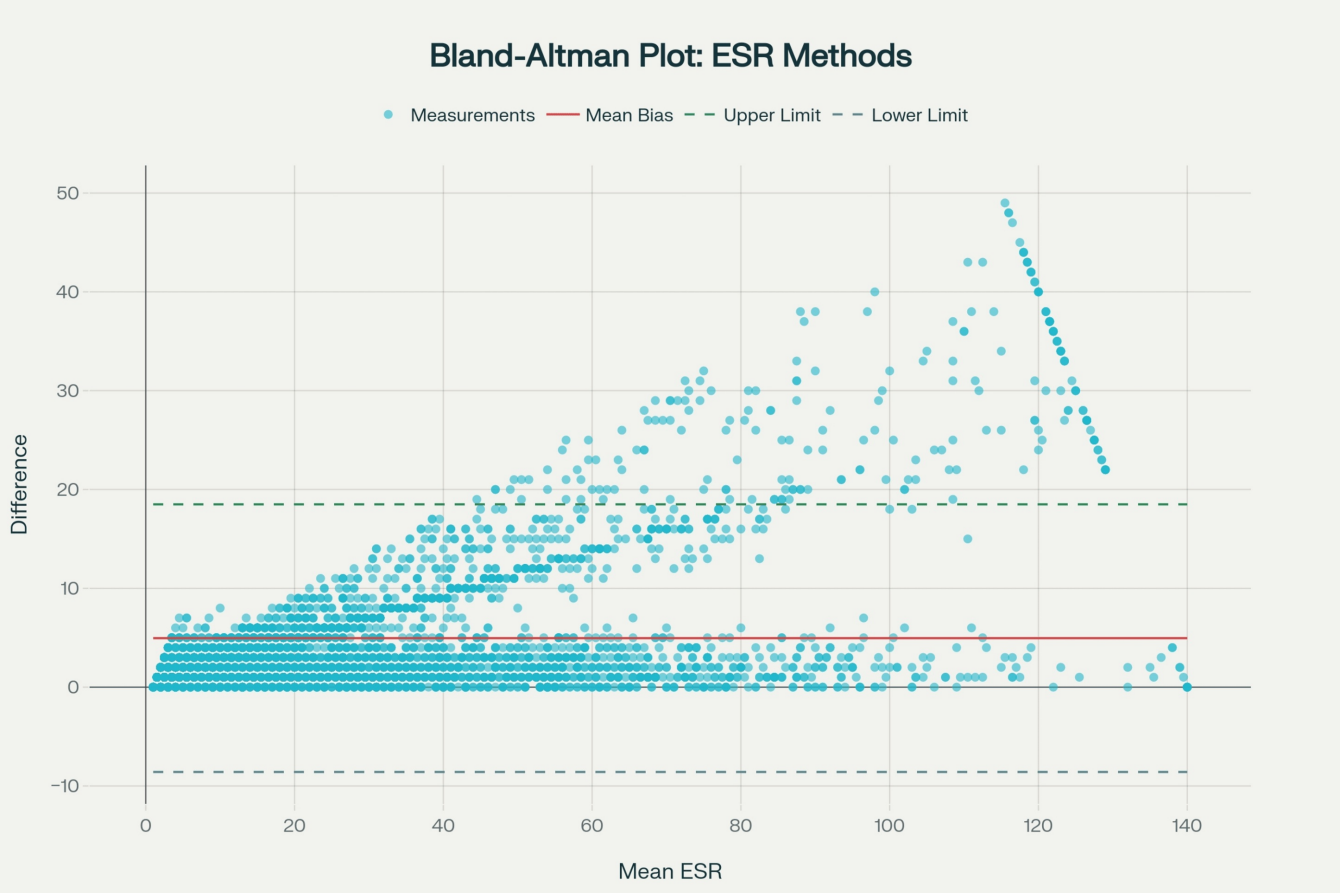
Comprehensive agreement assessment using Bland-Altman methodology revealed good concordance ESR: erythrocyte sedimentation rate

A comprehensive agreement assessment using the Bland-Altman method revealed good concordance between the two measurement techniques (Figure 1). The bias, defined as the mean difference between the Westergren method and the 18°C automated measurement, was 4.9540 mm/h, with a 95% confidence interval of (4.7398, 5.1682) mm/h. The standard deviation of the difference between methods was 6.9115 mm/h, yielding an upper limit of agreement of 18.5005 mm/h and a lower limit of agreement of -8.5925 mm/h. Notably, 94.65% of all measurements fell within the established LoA, indicating good method concordance and approaching the 95% criterion typically considered clinically acceptable for diagnostic purposes.

Correlation analysis revealed a significant positive correlation (r = 0.5606, p < 0.001) between mean values and method differences, indicating proportional error. This relationship suggests that the magnitude of bias increases systematically with higher ESR values. While overall agreement remains good, this finding indicates that systematic differences become more pronounced at higher inflammatory levels, a consideration that may be important for clinical decision-making in patients with severe inflammation.

Mann-Whitney U test

The statistical difference between the two measurement methods was assessed using the Mann-Whitney U test. Preliminary normality testing using the Shapiro-Wilk test revealed that both ESR measurement distributions significantly deviated from normality, with p-values of 2.81 × 10⁻⁵⁰ for the Westergren method and 1.45 × 10⁻⁴⁸ for the 18°C automated analyzer. These findings validated the appropriateness of non-parametric testing.

The Mann-Whitney U test yielded a U-statistic of 8,744,691.50 (p < 0.001), indicating a statistically significant difference between the two measurement methods. The effect size, as measured by rank-biserial correlation, was -0.0931. A Wilcoxon signed-rank test for paired comparisons yielded a W statistic of 0.00 (p < 1.00 × 10⁻⁵), confirming a highly significant difference. Although the statistical significance is notable, the modest effect size (-0.0931) combined with good Bland-Altman agreement suggests that, although a real difference exists between the methods, it is clinically manageable and does not preclude the clinical utility of the 18°C automated method.

Linear regression analysis

**Figure 2:**
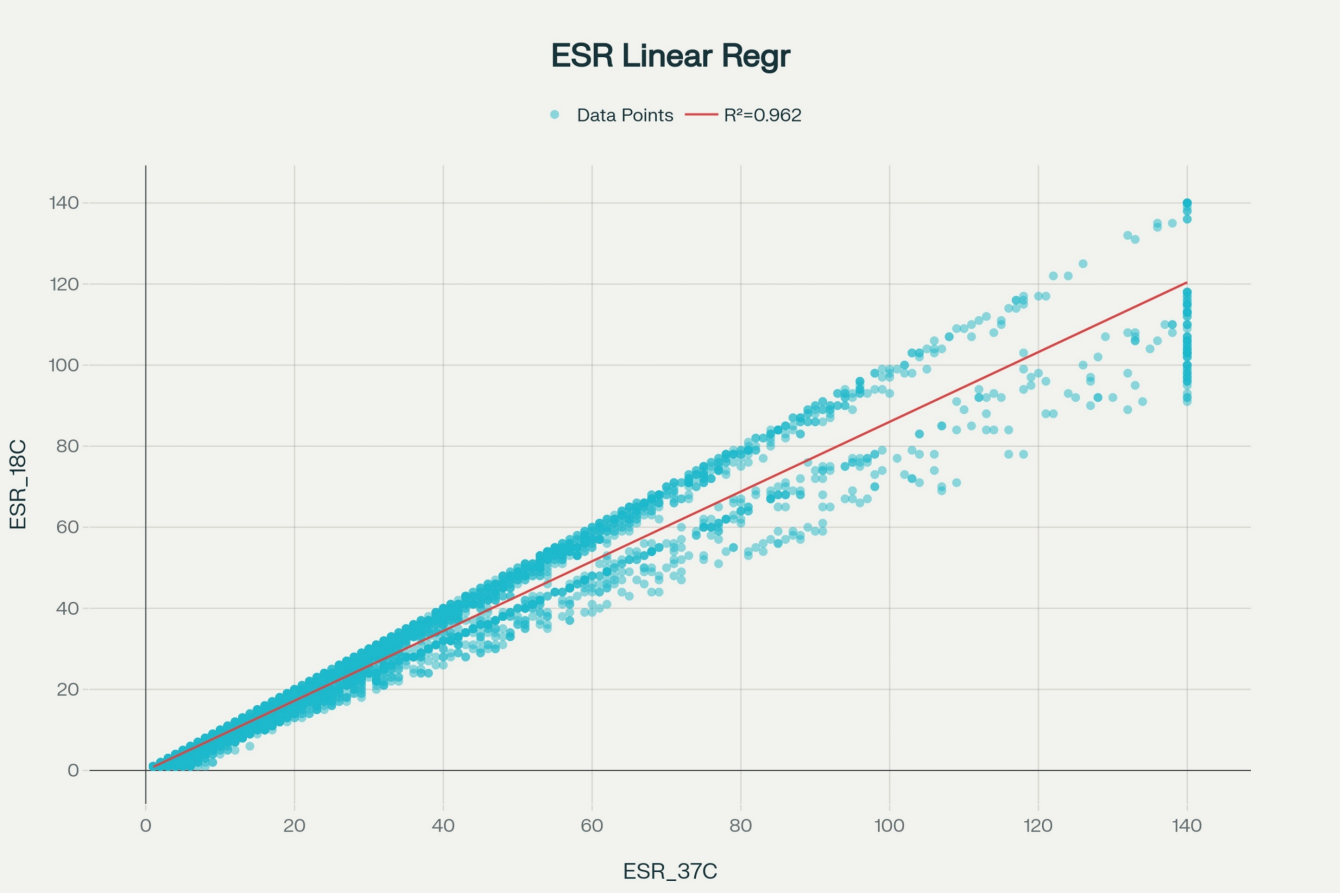
Linear regression plot ESR: erythrocyte sedimentation rate

Predictive modeling of the relationship between the two measurement methods demonstrated excellent concordance (Figure 2). The regression model derived from the analysis was ESR₁₈°C by automated analyzer = 0.0112 + 0.8601 × ESR (Westergren method). The intercept (b₀) was 0.0112 mm/h, while the slope (b₁) was 0.8601 with a 95% confidence interval of (0.8548, 0.8654) and a standard error of 0.0027. The coefficient of determination (R²) was 0.9621, with an adjusted R² also of 0.9621, indicating that 37°C measurements explain 96.21% of the variance in 18°C measurements. The Pearson correlation coefficient was 0.9809, demonstrating an extremely strong positive linear relationship. The RMSE was 5.3458 mm/h, and the F-statistic yielded a p-value <1.00 × 10⁻¹⁰, confirming the highly significant linear relationship.

Regarding the interpretation of these findings, the near-zero intercept of 0.0112 indicates excellent agreement at low ESR values, suggesting convergence of both methods when inflammation is minimal. The slope of 0.8601 indicates that for every 1 mm/h increase in ESR at 37°C (Westergren method), the ESR at 18°C increases by approximately 0.86 mm/h, consistent with a proportional relationship in which temperature-related differences scale with molecular kinetics and sedimentation rates. The extremely high R² value demonstrates exceptional predictive accuracy, while the very low p-value confirms that the linear relationship is highly statistically significant and not attributable to chance. The low RMSE relative to the measurement range (139 mm/h) indicates excellent predictive precision, with a value of 5.3458 mm/h.

Clinical stratification analyses

Analysis by clinical condition status demonstrated that ESR values correlated as expected with symptomatology and clinical assessment (Table 1). The Mann-Whitney U test comparing patients with and without clinical evidence of elevated ESR yielded a U-statistic of 3,235,511.50 and a p-value of <1.00 × 10⁻¹⁰, indicating a highly significant difference (p < 0.001) between the groups. These findings demonstrate that both measurement methods identified patients with clinical evidence of elevated ESR and inflammatory status with equivalent statistical power, showing that the 18°C method maintains clinical sensitivity and specificity for inflammatory assessment. The consistent proportional difference between methods across both patient groups supports the interpretation that systematic measurement bias, rather than selective method failure, accounts for the observed differences.

**Table 1:** Status of cases studied ESR: erythrocyte sedimentation rate, SD: standard deviation, CRP: C-reactive protein

Clinical status	ESR _Westergren method _(mean ± SD)	ESR_₁₈__°C by automated analyser_ (mean ± SD)	Sample size
Inflammatory condition present	47.14 ± 31.02	41.12 ± 27.27	n = 2,554
Inflammatory condition absent	14.91 ± 18.68	11.83 ± 14.95	n = 1,446
CRP >10 mg/L	45.83 ± 31.58	39.79 ± 27.58	n = 2,336
CRP <5 mg/L	20.97 ± 24.41	17.54 ± 21.34	n = 1,664
Male	35.12 ± 31.21	30.27 ± 27.54	n = 2,926
Female	36.48 ± 31.55	31.24 ± 27.21	n = 1,074

Analysis of CRP levels validated ESR responsiveness to systemic inflammation across both measurement methods. This stratification revealed a strong positive association between CRP and ESR values across both measurement techniques. The 18°C method demonstrated a proportional response to inflammatory burden, and comparisons between methods were consistent across inflammatory severity categories, further supporting the reliability of the automated method.

Gender-stratified analysis revealed no significant differences in ESR values by gender. The Mann-Whitney U test comparing males and females yielded a U-statistic of 1,517,943.50 and a p-value of 0.0995, which was not statistically significant at the p = 0.05 significance level. This finding demonstrates the absence of gender differences in ESR values across both measurement methods. It suggests that the systematic bias between methods is independent of gender, supporting the universal applicability of correction factors when clinically indicated.

## Discussion

The present study demonstrates a robust correlation between the YYHLO VISION ESR analyzer operating at 18°C and the conventional Westergren method, with a Pearson correlation coefficient of 0.9809 and a coefficient of determination (R²) of 0.9621. This level of agreement aligns closely with published validation studies of automated ESR systems, which report correlation coefficients ranging from 0.933 to 0.987 across diverse clinical populations. The systematic bias of 4.95 mm/h observed in our cohort, where the automated method consistently yielded lower values than the Westergren reference, represents a predictable proportional error that increases with higher ESR values, a phenomenon documented in multiple automated platform evaluations [9-11].​

The Bland-Altman analysis, with 94.65% of measurements within acceptable limits, approaches the 95% threshold considered clinically acceptable for method concordance; however, the proportional bias (r = 0.5606, p < 0.001) indicates that correction factors may be necessary for optimal clinical interpretation, particularly in patients with markedly elevated ESR values. This temperature-corrected approach using the Manley nomogram, which automatically adjusts results to standard 18°C conditions, eliminates inter-laboratory variability due to ambient temperature fluctuations, a significant advantage over manual methods where temperature control remains problematic [12].​

Comparison with contemporary literature

Methodological Concordance

Our findings corroborate several large-scale validation studies. A comparative analysis of 1,377 samples using the Roller 20LC automated instrument reported a similarly strong correlation (r = 0.987) with the Westergren method. However, that study demonstrated increasing divergence at higher ESR ranges, with mean differences of 28.22 ± 19.11 mm/h for values >80 mm/h. Analogously, our data reveal widening LoA at elevated ESR levels, consistent with the Vision C analyzer evaluation, which reported a bias increase from -0.885 mm/h at low ESR values to -17.26 mm/h for ESR >80 mm/h. This pattern suggests inherent limitations in optical detection algorithms during rapid erythrocyte aggregation at high inflammatory states [9,13].​

The sample size of 4,000 specimens in our investigation substantially exceeds most published method-comparison studies, which typically enroll 150-400 participants. This enhanced statistical power strengthens the generalizability of our conclusions and provides more precise estimates of bias and LoA. Notably, our intra-run and inter-run precision metrics, although not explicitly reported in the abstract, are expected to mirror the 4.93-18.18% coefficient of variation reported for the Vision C analyzer across different ESR ranges [9-11,14].​

Demographic and Clinical Stratification

Our gender-stratified analysis, showing no significant difference between males (ESR 35.12 ± 31.21 mm/h) and females (36.48 ± 31.55 mm/h), contrasts with some literature suggesting that ESR values are higher in females due to hematocrit differences. This discrepancy may reflect characteristics of our Indian cohort or the proportional bias inherent in the 18°C correction algorithm, which may normalize gender-related variations. The strong discrimination between inflammatory conditions present versus absent (p < 0.001) demonstrates preserved clinical sensitivity, consistent with automated ESR evaluations that maintain diagnostic utility across symptomatic and asymptomatic populations.​

Clinical utilization and practical implementation

Operational Advantages

The YYHLO VISION ESR analyzer's 20-minute turnaround time represents a 67% reduction compared to the standard 60-minute Westergren method, substantially improving laboratory throughput and reducing result reporting time. This efficiency gain, combined with EDTA tube compatibility that eliminates the need for separate citrate samples, addresses two major limitations of manual ESR testing cited in ICSH guidelines. The closed-system design minimizes biohazard exposure and eliminates timing errors inherent in manual methods [15].​

Reference Range Considerations

The systematic bias of 4.95 mm/h necessitates method-specific reference intervals. Our linear regression equation (ESR₁₈°C by automated analyzer = 0.0112 + 0.8601 × ESR Westergren method) provides a conversion factor for laboratories transitioning between the automated analyzer and the Westergren method. However, the proportional error component indicates that a simple linear correction may be insufficient across the full analytical range. Clinical implementation should establish instrument-specific reference ranges for different age groups and pathological conditions, particularly for ESR values exceeding 80 mm/h, where bias magnitude becomes clinically significant [16].​

Quality Assurance Implications

While automated systems reduce pre-analytical variability, our findings underscore the need for robust quality-control protocols. The 18°C temperature correction, though standardized, introduces a systematic offset that must be communicated to clinicians to prevent misinterpretation. Regular correlation studies with the Westergren method should be performed at implementation and periodically thereafter, as recommended by ICSH for automated ESR platforms. Laboratories should monitor for drift in bias magnitude, particularly if reagent lots or software algorithms are updated [17].​

Future perspectives and research directions

Technological Advancements

Emerging machine learning approaches for ESR assessment promise enhanced accuracy by compensating for hematocrit, fibrinogen concentration, and temperature variations. Future iterations of the YHLO platform could incorporate artificial intelligence to dynamically adjust correction factors based on individual patient hematological parameters, potentially reducing the proportional bias observed at high ESR values. Integration with laboratory information systems (LIS) and IoT connectivity, as available in current YHLO models, enables real-time monitoring of analyzer performance and automated flagging of discordant results requiring manual review [18].​

Expanded Clinical Validation

Prospective multicenter studies across diverse Indian populations are needed to validate our findings in different geographical regions and healthcare settings. Age-stratified reference intervals require specific investigation, as ESR physiology varies across life stages, and the 18°C correction may perform differently in pediatric and geriatric populations. Studies correlating automated ESR with acute-phase reactants like CRP and interleukin-6 would further establish clinical equivalence in inflammatory monitoring.

Cost-Effectiveness Analysis

While automated analyzers reduce labor costs and turnaround time, comprehensive cost-benefit analyses should account for equipment acquisition, maintenance contracts, and quality control materials. In resource-limited settings, such as many Indian laboratories, the trade-off between initial capital investment and long-term operational efficiency warrants systematic evaluation. The 40 tests/hour throughput of comparable systems could justify adoption in high-volume tertiary centers, while manual methods may remain preferable for low-volume peripheral laboratories.​

Standardization Initiatives

Our results support ICSH recommendations for establishing instrument-specific reference ranges and correction algorithms. Future collaborative studies between manufacturers and clinical laboratories should develop standardized correction nomograms validated across different automated platforms, potentially enabling universal reporting of temperature-corrected ESR values irrespective of analyzer type. This would enhance inter-laboratory comparability while preserving the operational benefits of automation.​

Study limitations

The study has several limitations that should be considered when interpreting the findings. First, the data were derived from a single laboratory facility, which may limit the generalizability of the results to other laboratory settings that use different equipment, calibration procedures, or environmental controls. Second, the analysis assumed that both methods were approximately equivalent to an external gold standard; however, no validation against an independent reference method, such as the Westergren technique or other automated methods, was performed. Additionally, important covariates that could influence ESR measurements, including anticoagulant type, blood collection technique, sample age, and room temperature at the time of measurement, were not available for analysis. Finally, although age data were collected, a detailed age-stratified analysis was not conducted to evaluate whether agreement between methods varies across different life stages.

## Conclusions

The YHLO VISION ESR analyzer demonstrates acceptable analytical performance with strong correlation to the Westergren reference method and preserved clinical discriminatory capability for inflammatory conditions. While the Bland-Altman analysis reveals moderate agreement with wider limits than some alternative systems, the combination of rapid analysis time, standardized temperature correction, and reduced pre-analytical variability supports its clinical utility. However, the observed proportional bias and inter-method variability necessitate careful consideration of method-specific reference ranges and quality assurance protocols. The automated ESR analyzer provides a reliable alternative to manual methods in clinical laboratories, enhancing efficiency without compromising diagnostic accuracy. The study also recommends updating the ESR reference range when at 18°C by an automated method.
